# Miniature Modular Reconfigurable Underwater Robot Based on Synthetic Jet

**DOI:** 10.1002/advs.202406956

**Published:** 2024-08-13

**Authors:** Dehong Wang, Fanheng Zhang, Shijing Zhang, Daqing Liu, Jing Li, Weishan Chen, Jie Deng, Yingxiang Liu

**Affiliations:** ^1^ State Key Laboratory of Robotics and System Harbin Institute of Technology Harbin 150001 China

**Keywords:** integrated system, modular reconfigurable robot, synthetic jet, underwater robot

## Abstract

Modular reconfigurable robots exhibit prominent advantages in the reconnaissance and exploration tasks within unstructured environments for their characteristics of high adaptability and high robustness. However, due to the limitations in locomotion mechanism and integration requirements, the modular design of miniature robots in the aquatic environment encounters significant challenges. Here, a modular strategy based on the synthetic jet principle is proposed, and a modular reconfigurable robot system is developed. Specialized bottom and side jet actuators are designed with vibration motors as excitation sources, and a motion module is developed incorporating the jet actuators to realize three‐dimensional agile motion. Its linear, rotational, and ascending motion speeds reach 70.7 mm s^−1^, 3.3 rad s^−1^, and 28.7 mm s^−1^, respectively. The module integrates the power supply, communication, and control system with a small size of 48 mm × 38 mm × 38 mm, which ensures a wireless controllable motion. Then, various configurations of the multi‐module robot system are established with corresponding motion schemes, and the experiments with replaceable intermediate modules are further conducted to verify the transportation and image‐capturing functions. This work demonstrates the effectiveness of synthetic jet propulsion for aquatic modular reconfigurable robot systems, and it exhibits profound potential in future underwater applications.

## Graphical abstract

A miniature modular reconfigurable underwater robot system adopting the synthetic jet as a propulsion mechanism is developed. The motion module fuses two types of jet actuators and realizes the integration of power supply, control, and communication functions. Various configurations between different motion and intermediate modules are designed, and the application potential of the robot system is verified by experiments.

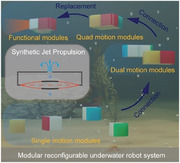

## 1 Introduction

Robots are indispensable in various aspects of modern society, from manufacturing to exploration. Conventional monolithic robots are typically designed for specific tasks or functions.^[^ ^1^, ^2^ ^]^ However, in unpredictable and unstructured environments, robots are required to possess diverse motion capabilities or functional traits to meet the demands of different tasks.^[^ ^3^, ^4^ ^]^ In addition, robots must be repaired promptly in case of failures to ensure reliability during missions. These requirements lead to an immediate need for robots with modular reconfigurable capabilities.

Modular reconfiguration is always a significant theme in robotic research.^[^ ^5^ ^]^ The modular reconfigurable robot system is inspired by cells,^[^ ^6^ ^]^ and comprises multiple homogeneous or specialized modules. Then, it can reconfigure the geometric structure, assembly state, as well as software connections.^[^ ^7^, ^8^, ^9^ ^]^ It generally possesses several distinct advantages, including high adaptability, high robustness, and low cost.^[^ ^10^ ^]^ The modules can reconnect the structure into more appropriate typologies or alter the capabilities of functional modules according to different requirements.^[^ ^11^, ^12^, ^13^ ^]^ The robustness benefits from the redundancy of the modules; the damage of a certain local module will not affect the function of the whole system, and a simple replacement with an interchangeable module can restore its function directly.^[^ ^14^, ^15^ ^]^ This characteristic also reduces the maintenance cost of the robot system. Besides, the modular design reduces the structural and functional complexity of the system, thereby reducing production costs.^[^ ^16^ ^]^

However, there are usually more strict demands for modular reconfigurable robots in underwater environments such as oceans or rivers.^[^ ^17^ ^]^ On the one hand, during the motion in narrow or shallow aquatic environments, the presence of reefs or corals will hinder the movement of the robots; and the cables or other connections will also limit the motion range. This proposes requirements for integrated underwater robots with small sizes. On the other hand, due to the restriction of propulsion methods and the sealing requirements in underwater environments, it is more complicated to implement modular designs for the robots. Thus, it is challenging to design such a miniature underwater robot system with reconfigurable modules and realize the tasks in a narrow and unstructured environment.

The existing underwater propulsion strategies mainly include conventional methods, such as propeller,^[^ ^18^, ^19^ ^]^ and bionic methods, such as body‐caudal fin (BCF),^[^ ^20^, ^21^, ^22^, ^23^ ^]^ median pair fin (MPF)^[^ ^24^, ^25^, ^26^, ^27^ ^]^ propulsion, and jet thruster.^[^ ^28^, ^29^, ^30^ ^]^ The propeller is generally utilized in autonomous underwater vehicles (AUV)^[^ ^17^, ^31^, ^32^ ^]^ with large size and weight, thus unsuitable for operation in narrow spaces. The BCF and MPF propulsions are inspired by the locomotion of fishes and are usually complicated in mechanism, which makes it difficult to realize modularization design and motion control. The jet thruster imitates the motion principle of the cephalopods,^[^ ^33^, ^34^ ^]^ such as jellyfish and squid, while the conventional thrust requires an independent inlet and outlet port, resulting in a complex structure.^[^ ^35^ ^]^ The synthetic jet, as a new jet propulsion method, only requires a single jet orifice and can achieve motion based on high‐frequency and small‐amplitude vibration.^[^ ^36^, ^37^ ^]^ An excitation source is utilized to drive a diaphragm to produce periodic motions; thus, the fluid inside the cavity can generate a series of vortex rings, which expand outward after passing through the small orifice and form a momentum jet.^[^ ^38^, ^39^ ^]^ Therefore, the synthetic jet method exhibits obvious advantages of compact structure, fast response, simple control scheme, and low cost of manufacture. These characteristics facilitate the realization of modularization and reconfiguration. However, in available research about synthetic jets, typical excitation sources usually contain piezoelectric materials,^[^ ^40^ ^]^ dielectric elastomers,^[^ ^41^ ^]^ and transmission mechanisms,^[^ ^42^ ^]^ which require high excitation voltages and specialized signals and cannot eliminate the constraints of a wiring harness and realize the integration of the control system.

**Figure 1:**
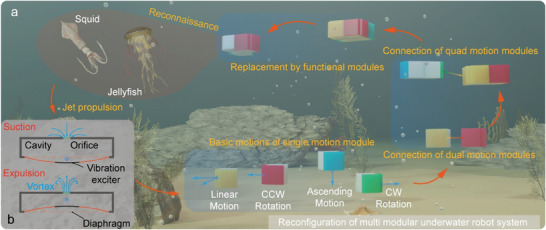
The synthetic jet principle and the combination schemes for the miniature modular reconfigurable underwater robot. a) Motion and combination schemes of the reconfigurable underwater robots. b) The suction and expulsion process of synthetic jet principles.

Aiming at the requirements in the aquatic environment and the existing problems of current propulsion methods, we propose a modularization solution based on the synthetic jet propulsion mechanism and develop a series of integrated modular reconfigurable robots, as shown in **Figure** **1**a,b. During the design of the whole modular reconfigurable robot system, we have carried out a comprehensive study in different layers: the synthetic jet actuator—the single motion module—the combination strategy—the application prospection. The main contributions of this work are as follows: 1) As for the synthetic jet actuators, we use the electromagnetic vibration motor as the excitation source for the first time to achieve synthetic jet propulsion underwater; and we design two types of actuators in the bottom and side jet directions based on the motors, which ensure the flexibility in the structure design of motion modules. 2) As for the single motion module, we arrange the jet actuators in X, Y, and Z directions, respectively, and realize the integration of the power supply, communication, and control system, which ensures the highly agile multi‐degree of freedom (DOF) self‐propulsion of a single module. The size of an individual module is only 48 mm × 38 mm × 38 mm. This integrated and miniaturized structure can realize various chain or lattice configurations ensuring expansion ability. 3) As for the multi‐module combination, we propose various configurations between different motion and intermediate modules along with their different motion strategies based on the cubic structure of the modules. It verifies the reconfigurable and interchangeable characteristics and extends the diversity of the robot system. 4) As for the application prospection, we have carried out preliminary explorations of possible applications and designed basic functional modules, such as transportation and image‐capturing modules, which enable the selection of specific configurations according to application requirements and demonstrate the potential of the modular robot systems. The combination and motion strategies of the modular robot system are shown in Movie S1 (Supporting Information). This work effectively demonstrates the application potential of synthetic jet propulsion in the miniature modular reconfigurable underwater robot system. It exhibits the advantages of high robustness and adaptability and is expected to play an important role in the tasks of detection and reconnaissance of the underwater environment in the future.

## 2 Synthetic Jet Mechanism and Design of the Synthetic Jet Actuators

**Figure 2:**
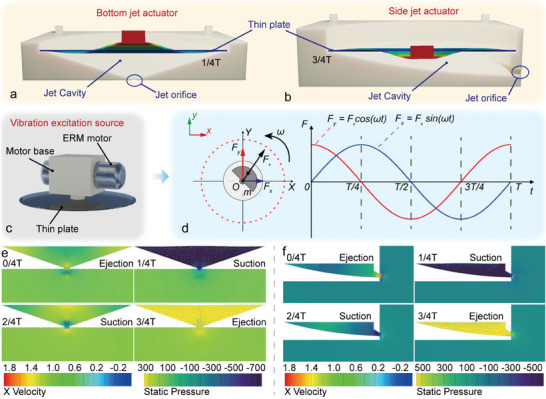
Synthetic jet mechanism and design of bottom and side jet actuator. a) Structure design of bottom jet actuator. b) Structure design of side jet actuator. c) Waterproof ERM motor connected with the thin plate. d) Centrifugal force generated by the eccentric mass. e) Flow field of bottom jet actuator acquired from fluid‐structure interaction simulation. f) Flow field of side jet actuator acquired from fluid‐structure interaction simulation.

The synthetic jet actuator is the propulsion component of the motion module, which determines the locomotion effect as well as the overall size of the modules. Therefore, the design of the synthetic jet actuator is critical for this modular reconfigurable robot system. As only one orifice is required, the structure of the synthetic jet can be compact and simple. We design two types of synthetic jet actuators to facilitate the realization of the multi‐DOF motion of a single‐motion module. The first type is a bottom jet actuator, as shown in **Figure** **2**a. It adopts a conical shape for the jet cavity and arranges the orifice on the bottom surface. Thus, the jet in the direction vertical to the bottom can be generated under the direct excitation of the vibration source. The other type is a side jet actuator, as shown in Figure 2b. It adopts an approximate oblique conical shape for the jet cavity and arranges the orifice on the side surface so as to generate a jet in the direction vertical to the side face.

Both types of synthetic jet actuators utilize a waterproof eccentric rotating mass (ERM) vibration motor as the excitation source, as shown in Figure 2c. The ERM motor is mounted on the center of a thin plate by a motor base and then fixed on the jet cavity together. The ERM motor can produce an elliptical vibration through the rotation of the eccentric mass under a direct current power supply, as shown in Figure 2d. The centrifugal force *F*(*t*) can be expressed as:

(1) $$\left\{\begin{array}{c} F\left(t\right)=F_{0}e^{i\omega t}\\ F_{0}=md\omega^{2} \end{array}\right.$$

where *F*_0_ is the amplitude of centrifugal force, *m* is the mass of the eccentric mass, *d* is the eccentric distance, and *ω* is the rotary velocity. The equation shows that the amplitude of the centrifugal force is in a quadratic relationship with the angular velocity as well as the vibration frequency. Besides, the angular velocity is positively related to the input voltage.

The direction of the centrifugal force is outward from the rotational center to the mass center of the eccentric mass, then the simple harmonic vibration in the horizontal and vertical directions can be obtained as:

(2) $$\left\{\begin{array}{c} F_{x}=F_{0}\cos\left(\omega t\right)\\ F_{y}=F_{0}\sin\left(\omega t\right) \end{array}\right.$$

where *F_x_* is the horizontal excitation force, and *F_y_* is the vertical excitation force.

The thin plate will produce different vibration shapes under the excitation of the ERM motor (see Figure S1, Supporting Information). It can be seen that the first and fourth modes will have stable suction and expulsion capability and can be applied to the synthetic jet actuation effectively, while the second and third modes cannot produce effective locomotion due to the compensation effect of their own deformations on the volume change.

The frequency of the ERM motor can reach ≈270 Hz in a free state according to experiment results, and it will decrease to ≈210 Hz when connected to the thin plates. Thus, a frequency match between the mode shape of the thin plate and the vibration motor should be conducted to ensure the effective jetting of the actuator. According to the simulation (see Figure S1d, Supporting Information), the dimension of the thin plate is selected as Φ30 mm × 0.25 mm, in which the fourth resonant frequency can be 228.35 Hz. Then, the fluid‐structure interaction simulation is carried out based on the selected thin plate, as shown in Figure 2e,f. With the deformation of the thin plate, a significant suction‐expulsion phenomenon, as well as a stable vortex ring motion, is generated near the jet orifice in one cycle. The development process of the synthetic jet is observed via a particle image velocimetry (PIV) method (see Figure S2a and Movie S2, Supporting Information). The net force of the jet actuator is measured to be ≈7 mN, which can produce an effective propulsion effect, as shown in Figure S2b (Supporting Information).

## 3 Design of Multi‐DOF Synthetic Jet Motion Module

**Figure 3:**
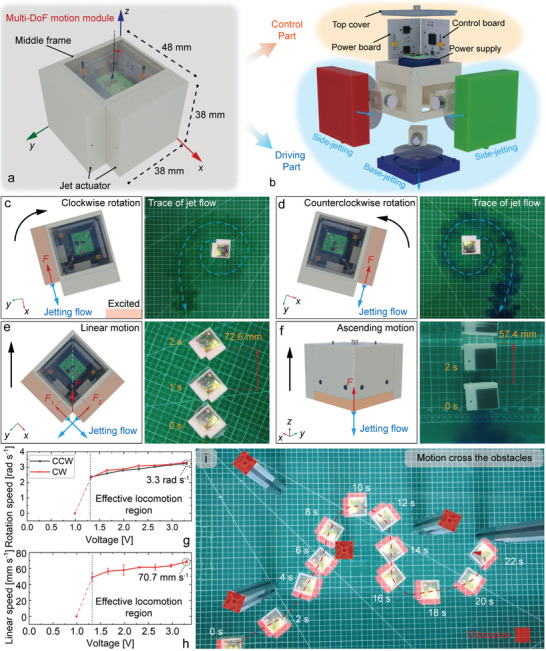
Synthetic jet motion module design and multi‐DOF motion schemes. a) Structure design of multi‐DOF motion module. b) Arrangement of jet actuators and control system in the motion module. c) Clockwise rotation method of the motion module. d) Counterclockwise rotation method of the motion module. e) Linear motion method of the motion module. f) Ascending motion method of the motion module. g) Rotational speed in two directions of the motion module at different voltages (Sample size n = 3, Mean ± SD). h) Linear motion speed of the motion module at different voltages (Sample size n = 3, Mean ± SD). i) Controlled motion of the motion module crossing the obstacles.

The motion module is expected to be designed with multi‐DOF self‐propulsion capability. Therefore, the bottom and side synthetic jet actuators are mounted onto a single motion module, as shown in **Figure** **3**a. This motion module adopts an approximate cubic appearance, which can facilitate the combination and replacement of multiple modules. The overall dimension of a single motion module is ≈48 mm × 38 mm × 38 mm, and the mass of the module is ≈79.0 g. The balance between the gravity and the buoyancy force is realized according to the structure design and component distribution. Then, the motion module can be suspended in water in a stable state (see Note S1 and Figure S3, Supporting Information).

Three jet actuators are fixed on the middle frame so that each motion module can produce motion in the *x*, *y*, and *z* directions, respectively, as shown in Figure 3b. A bottom jet actuator is arranged at the base for the ascending motion of the module, while two side jet actuators are arranged on the two neighboring perpendicular surfaces for the planar motion. This arrangement of the jet actuators guarantees motion flexibility and ensures that all jet effects will not interfere as connected with other modules. The electronic system for power supply, control, and communication is fully integrated inside the module to realize remote control (see Note S2 and Figure S4, Supporting Information for detailed information).

When a unilateral side jet actuator is working, it can produce a propulsion force along this side edge. The force will create a rotational moment around the mass center of the motion module, causing the entire module to undergo a clockwise (CW) or counterclockwise (CCW) in‐situ rotation, as shown in Figure 3c,d. When both side jet actuators work simultaneously, the jet flows from the two side jet actuators merge together, thus generating a linear force along the bisector direction. It will propel the motion module to produce a linear motion, as shown in Figure 3e. The fluid‐structure interaction simulation for the linear motion case is shown in Figure S5. The maximum rotational speed of the motion module is ≈3.3 rad s^−1^, and the maximum linear speed is ≈70.7 mm s^−1^, as shown in Figure 3g,h. The linear and rotational speeds are maintained in a stable motion state within the voltage range of 1.3–3.3 V and exhibit a linearly decreasing trend. As the exerted voltage decreases to ≈1.0 V, the motion speed will decrease instantly to almost 0. The main reason is that the thin plate will not be able to produce stable suction‐expulsion deformation under the excitation of the motors at this voltage, and it cannot maintain the motion of the module.

The descending and ascending motion of this module is realized by a single bottom jet actuator, as shown in Figure 3f. As the actuator is excited, it will propel the motion module upward, and its ascending motion speed can reach up to 28.7 mm s^−1^. The motion module will automatically descend gradually when the bottom jet actuator is off. In addition, we also tested the controlled motion capability of this motion module, as shown in Figure 3i. It can be seen that the cooperation of these jet actuators realizes an agile motion, and the module can bypass the obstacles and reach the target location in a controlled state. The method to ensure the linear motion and regulate the motion orientation is illustrated in Note S3 (Supporting Information). The experiment videos of the single motion module are shown in Movie S3 (Supporting Information).

We conduct experimental evaluations about the endurance of the single motion module. The exciting voltages of the jet actuators are set at 3.3 V during the experiment. The capacity of the battery is ≈200 mAh. When a single jet actuator is working, the endurance of the module is ≈85 min; when two jet actuators are working simultaneously, the endurance is ≈50 min; and when three jet actuators are working simultaneously, the endurance is ≈30 min. Due to the design of the voltage stabilization unit, the driving performance during the whole process is relatively stable, and a sudden decrease occurs at the final stage, as shown in Figure S6 (Supporting Information). This endurance is well‐performed in such a small size. It should be noted that the specific endurance is directly related to the capacity of the battery; thus, a balance between the capacity and the module size can be weighted to increase the endurance in practical applications.

## 4 Configuration Strategies of Different Modules

The kinematic characteristics of a single motion module have been evaluated in detail. Then, a single multi‐DOF motion module can be connected with other homogeneous motion modules or specific intermediate modules to form a modular reconfigurable robot system, which can possess more motion and function capabilities. As for the coupling method between multiple modules, the magnetic coupling method is usually an effective solution for microrobots, which is simple and compact in structure. In addition, the magnetic connection has a good self‐alignment capability. It can simplify the docking process and contribute to accuracy. The reliability of the connection is also evaluated, as illustrated in Note S4 (Supporting Information).

**Figure 4:**
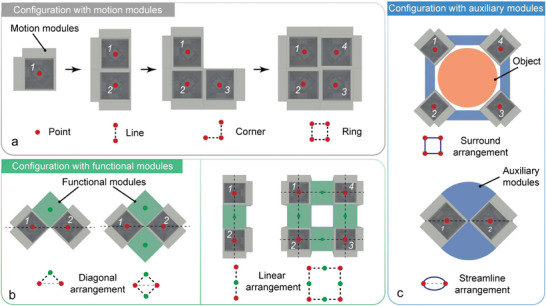
Configuration strategies between different modules. a) Basic configurations between homogeneous motion modules. b) Basic configurations between the diagonal‐arranged and linear‐arranged motion modules with functional modules. c) Basic configurations between the motion modules with auxiliary modules.

For the connection between homogeneous motion modules, the basic configurations are designed with two, three, and four motion modules to realize an expansion from point into line, corner, and ring, as shown in **Figure** **4**a. Each of the modules is integrated and capable of multi‐DOF propulsion. These configurations will exhibit various motion schemes, thereby increasing the agility of the entire robot system. The addition of motion modules increases the redundancy of the robot system and also improves the robustness of the overall system. The replacement process of the damaged motion module is shown in Figure S7 (Supporting Information).

Due to the regular structure and simple coupling interface of the motion module, it can be connected with other intermediate modules directly, except for the connection with each other. The intermediate modules can be functional modules to realize some specific functions or can be auxiliary modules to modify the typology patterns and realize the function expansion. Therefore, the final configurations of the combined robot systems vary depending on the design of the intermediate modules, which provide wider application ranges to the modular robots. We showcase two basic types of configurations with simple cubic intermediate modules in Figure 4b, which can be identified as diagonal and linear arrangements according to the distribution of motion modules. The diagonal arrangement is more compact, while the linear arrangement shows better expansion properties. The combinations with auxiliary modules are shown in Figure 4c. For smooth surface objects such as spheres or cylinders, the motion modules can combine with some auxiliary modules to form a ring and achieve transportation. They can also streamline their own shape by the auxiliary modules, thus improving their motion performance. As for the intermediate modules with different shapes and sizes (such as cuboid or prism), the configurations are shown in Note S5 and Figure S8 (Supporting Information).

## 5 Configurations Between Homogeneous Motion Modules

The combinations of the motion modules can improve the agility of the robot system and exhibit various motion DOFs. To analyze the motion characteristics of the basic configurations between homogeneous motion modules, we analyze two typical cases with two and four modules experimentally. In addition, we also support the reconfiguration of multiple modules at the software control level and develop a control software with multi‐connection and selective communication capabilities. Thus, it can send commands to selected modules, ensuring simultaneous control of multiple modules (see Figure S4, Supporting Information).

### 5.1 Locomotion Strategies of Dual Motion Modules

**Figure 5:**
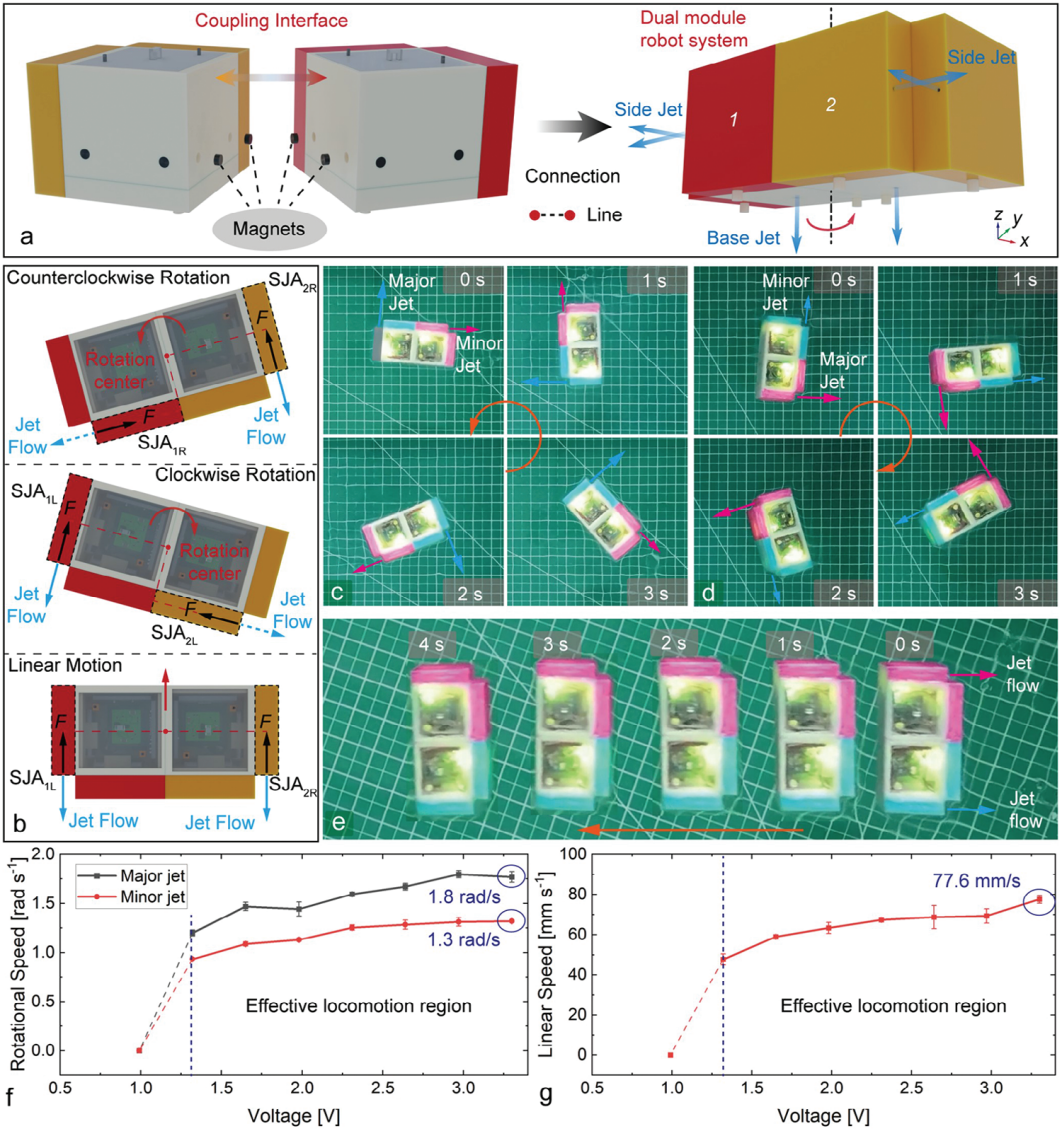
Connection between dual motion modules and the motion schemes. a) Connection method between two motion modules. b) Motion strategies of the dual modules. c) Counterclockwise rotational motion of the two modules. d) Clockwise rotational motion of the two modules. e) Linear motion of the two modules. f) Rotational motion speed excited by two jet orifices (Sample size n = 3, Mean ± SD). g) Linear motion speed of the two modules (Sample size n = 3, Mean ± SD).

The dual module configuration is shown in **Figure** **5**a, where two small permanent magnets are arranged on the non‐jet middle frame surfaces, allowing a direct connection between motion modules as a line. The combined robot system has two bottom jet actuators (BJAs) and four side jet actuators (SJAs).

This dual module combined configuration shows more agile motion capability in the water compared to the single motion module, as shown in Figure 5b. As either of the side jet actuators) SJA_1R_ or SJA_2R_ is excited, the combined robot system can produce counterclockwise rotational motion, as shown in Figure 5c. Conversely, as the side jet actuators SJA_1L_ or SJA_2L_ are excited, the robot system is able to produce clockwise rotational motion, as shown in Figure 5d. Typically, the moments produced by these two side jet actuators are different due to the arm lengths, which can be identified as major (SJA_1L_ and SJA_2R_) and minor (SJA_1R_ and SJA_2L_) jet actuators, respectively. Then, the combined robot system exhibits rotational motion capabilities with two stable stages when different jet actuators are excited individually, as shown in Figure 5f. It can be seen that as the major actuator is excited, the rotational speed is ≈1.8 rad s^−1^, and as the minor actuator is excited, the rotational speed is about only 1.3 rad s^−1^. The difference in the rotational speed remains relatively stable at different voltages.

When the side jet actuators SJA_1L_ and SJA_2R_ are excited at the same time, the combined robot system will produce linear motion, as shown in Figure 5e. Besides, the minor jet actuators can be used to adjust the motion directions and resist lateral disturbances. The maximum motion speed is ≈77.6 mm s^−1^, which is comparable to the motion speed of a single module. Similarly, the motion speed exhibits a steady decline state and drops abruptly at the voltage of ≈1.0 V, as shown in Figure 5g.

### 5.2 Locomotion Strategies of Quad Motion Modules

**Figure 6:**
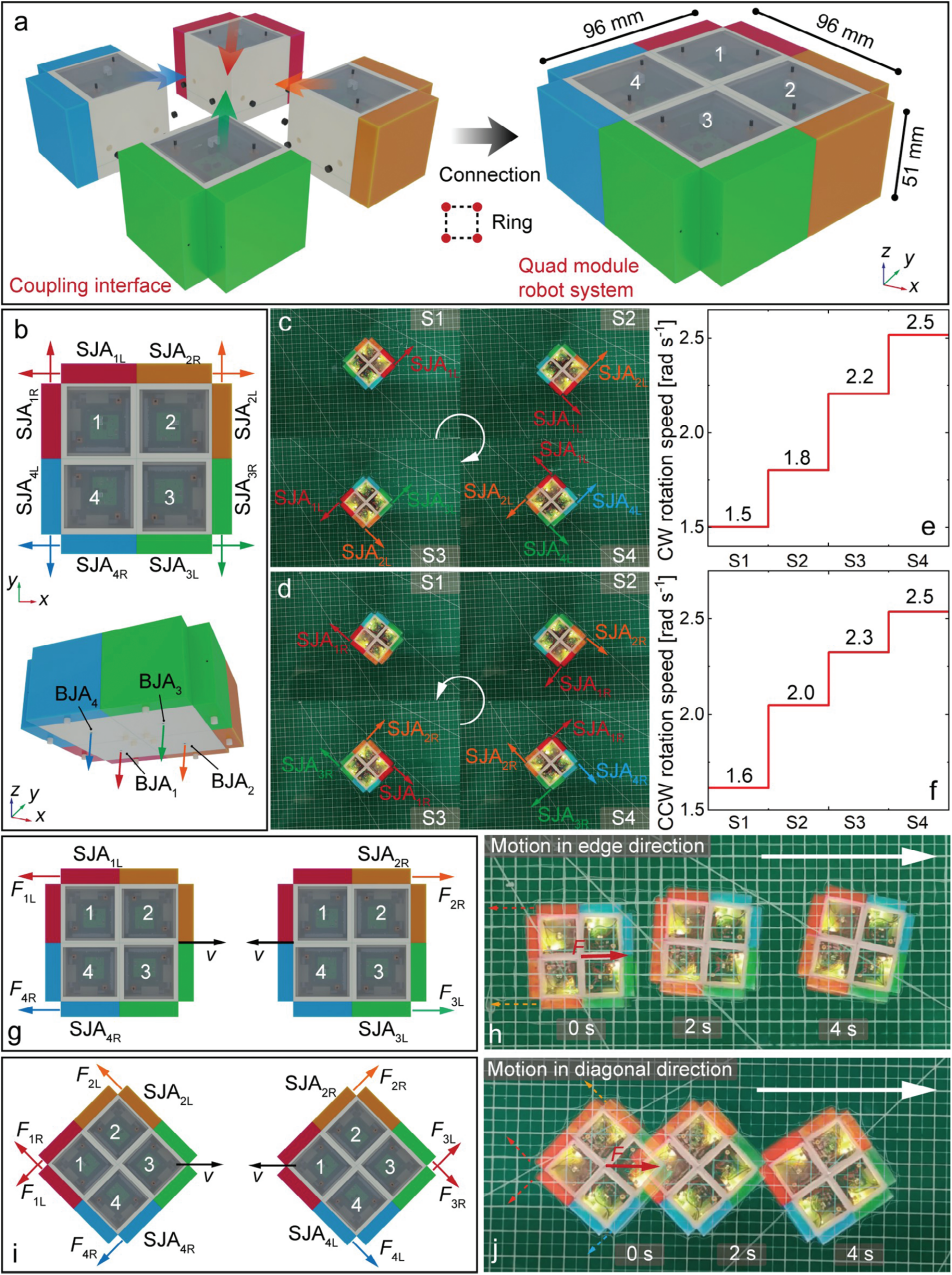
Motion experiments of quadra motion modules. a) Connection method between four motion modules. b) Distribution of the 12 jet actuators for the quad module combination. c) Clockwise rotational motion of the quad modules. d) Counterclockwise rotational motion of the quad modules. e) Four rotational speed stages of the clockwise rotational motion. f) Four rotational speed stages of the counterclockwise rotational motion. g) Linear motion scheme in the edge direction. h) Linear motion experiment in the edge direction. i) Linear motion scheme in the diagonal direction. j) Linear motion experiment in the diagonal direction.

The dual module configuration increases the agility in rotational motion, while there is no improvement for the linear motion scheme. The quad module configuration will enhance the motion schemes of the entire combined robot system significantly. The motion modules also use permanent magnets for connection, and the size of the connected robot system is about 96 mm × 96 mm × 51 mm, as shown in **Figure** **6**a. The whole robot system has four bottom jet actuators (BJA_1_‐BJA_4_) and eight side jet actuators, which guarantees its high agility and high robustness. All the jet actuators are marked in Figure 6b.

Benefiting from the well‐balanced configuration of the quad motion modules, each side jet actuator contributes equally to the rotational motion. With the combination of the eight side jet actuators, this quad‐module combined robot system can realize the clockwise and counterclockwise rotational motion at four stable states (S1–S4) based on the number of excited actuators, as shown in Figure 6c–f. The rotational speed is ≈1.6 rad s^−1^ under the propulsion of a single jet actuator, and it reaches 2.5 rad s^−1^ under the simultaneous propulsion of four actuators. It means that the failure of local actuators will only cause a decrease in performance but not affect the rotational motion capability.

As for the cases of linear motions, the combined robot system can freely move back and forth in the plane along all the edge directions and the diagonal directions flexibly. When both sets of jet actuators on one side (such as SJA_1L_ and SJA_4R_, SJA_3L_ and SJA_2R_) are activated simultaneously, it can produce linear motion along the edge directions with a capability for reverse motion, and the motion speed is ≈81.4 mm s^−1^, as shown in Figure 6g,h. The linear motions along the angular bisector directions exhibit two motion states. The simultaneous propulsion of the diagonally symmetric actuators (such as SJA_2L_ and SJA_4R_) or propulsion of a single corner module (such as SJA_1L_ and SJA_1R_) can both produce a diagonal linear motion, and the speed can be ≈63.0 and 62.1 mm s^−1^, respectively, as shown in Figure 6i,j. In addition, the linear motion trajectories of the quad motion module system are evaluated in all eight directions as different combinations of jet actuators are excited, as shown in Figure S9 (Supporting Information). The results demonstrate the high motion agility of the modular robot system.

It is noteworthy that the plethora of multi‐modules will not improve the performance necessarily but may lead to a reduction of motion parameters of the robot system, due to the increase in mass and resistance. However, the stability and robustness of the combined modules greatly compensate for this defect. The four module configurations only need at least two side jet actuators to realize the complete rotational and linear motion functions in the plane. Thus, the configurations of the combined modules should be decided based on the actual application and functional requirements.

## 6 Experiments of Configurations With Functional Modules

The advantages of the modular reconfigurable robot are not fully represented by the combination of homogeneous motion modules. Different structural designs of the intermediate modules can significantly expand the combination schemes and application potential due to reconfigurability. Therefore, we verify its basic functions, such as transportation and detection in water, utilizing simple cubic intermediate modules of similar size to the motion modules, as shown in Figure 4b.

**Figure 7:**
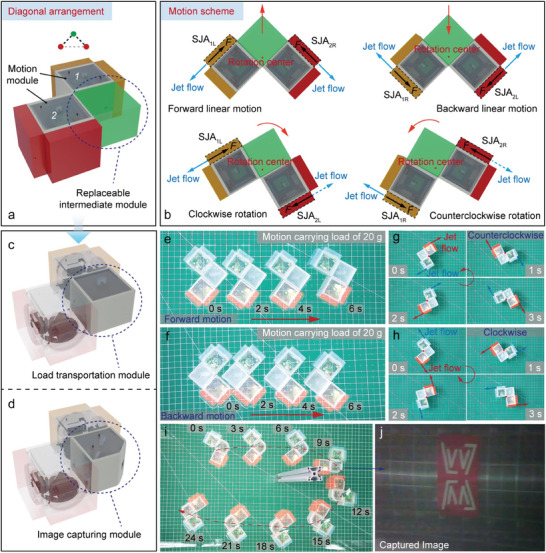
Motion schemes and experiments of diagonal configuration. a) Diagonal configuration with a replaceable intermediate module. b) Motion strategies of the diagonal configuration. c) Transportation module structure design. d) Image capturing module structure design. e) Forward linear motion when carrying the transportation module. f) Backward linear motion when carrying the transportation module. g) Counterclockwise rotational motion when carrying the transportation module. h) Clockwise rotational motion when carrying the transportation module. i) Motion trajectory when carrying the image‐capturing module. j) The frame of the captured video by the image capturing module.

The dual motion modules are utilized for the propulsion of the interchangeable intermediate modules, and the diagonal configuration is adopted for its flexibility, as shown in **Figure** **7**a. The intermediate module is arranged in the middle with dual modules on each side like an arrowhead. The motion modules can carry the intermediate module to produce forward linear motion when the side jet actuators SJA_1L_ and SJA_2R_ work simultaneously and produce reverse linear motion when the side jet actuators SJA_1R_ and SJA_2L_ work simultaneously, as shown in Figure 7b. Similarly, when the side jet actuators SJA_1L_ and SJA_2L_ are excited simultaneously, the combined robot system produces a clockwise rotational motion, while excitation of the side jet actuators SJA_1R_ and SJA_2R_ produces a counterclockwise rotational motion.

For this diagonal configuration, we designed two types of functional modules to evaluate the application potential, including the object transportation module and the image capturing module, as shown in Figure 7c,d. In the object transportation experiment, we placed a standard weight of 20 g inside the transportation module. The combined robot system realized the forward and backward linear motion under the propulsion of the dual motion modules with a maximum linear speed of 46.8 and 44.9 mm s^−1^, respectively, as shown in Figure 7e,f. It also realizes the clockwise and counterclockwise rotational motion with a maximum speed of 1.8 and 1.5 rad s^−1^, as shown in Figure 7g,h. In the image‐capturing experiment, the transportation module is replaced with an image‐capturing module in the diagonal configuration. The combined robot system is controlled to go around the obstacle and return, and the image‐capturing module records during the whole process. The motion trajectory in the water is shown in Figure 7i, and the frame at 9 s of the captured video is shown in Figure 7j.

In addition, we also adopt the basic linear configuration in Figure 4b for the transportation experiments. The linear configuration cannot realize the backward linear motion, but it can go through a narrow channel due to its slender size (See Note S6 and Figure S10, Supporting Information).

## 7 Conclusion

In this work, a miniature modular reconfigurable underwater robot system is developed using the synthetic jet as the propulsion mechanism. For the jet actuators, we adopt the ERM motor as the excitation source for the first time and design two types of synthetic jet actuators in aquatic environments for the bottom and side propulsion separately. For the motion modules, we combine two types of jet actuators to design an aquatic motion module with multi‐DOF propulsion capability and then realize the integration of power supply, control, and communication functions. For the combined robot system, we design various configurations with different motion or intermediate modules; and determine the motion schemes for these module configurations. For the robot applications, we design the basic functional modules and conduct experiments to verify the application potential of the modular reconfigurable robot system.

This work demonstrates the advantages of the synthetic jet principle as a propulsion method in the modularization and miniaturization design of underwater robots. On the one hand, this synthetic jet actuation does not require a complex propulsion mechanism, thus facilitating the modular design and connection with other modules. On the other hand, its excitation scheme is simple, and its jet structure is compact, which makes it convenient to realize the integration of the entire robot in a small size. Besides, we make an extensive comparison of the motion speed with other underwater robots, as shown in Note S7 and Figure S11 (Supporting Information).^[^ ^18^, ^21^, ^22^, ^23^, ^25^, ^26^, ^40^, ^41^, ^43^, ^44^, ^45^, ^46^, ^47^, ^48^, ^49^, ^50^, ^51^, ^52^, ^53^, ^54^, ^55^, ^56^, ^57^, ^58^, ^59^, ^60^, ^61^, ^62^, ^63^, ^64^, ^65^, ^66^, ^67^, ^68^, ^69^, ^70^ ^]^ The motion module in this work exhibits superior motion performance in terms of both size and motion speed.

This work reflects all the characteristics of a modular reconfigurable robot system sufficiently. As for robustness, due to the redundancy of multi‐module combinations, the failure of a single module hardly affects the function of the whole robot system, which only results in a decrease in parameters. Besides, the replacement ability of an arbitrary motion module ensures the reliability of the entire robot system during application. As for adaptability, this modular robot system exhibits various configurations to adapt to different motion environments through the combinations of different modules. The functional modules can also be replaced based on requirements only if the consistency of the coupling interface is ensured. As for the cost, the motion modules are fabricated by 3D printing, which decreases the cost of a single motion module, and the replacement also guarantees the cost of system maintenance.

With respect to the motion modules, the designed bottom and side jet actuators themselves are two kinds of sub‐modules essentially, which also have certain combination and replacement characteristics. These two types of jet actuators will be able to achieve a reconfiguration on the center frame of a single motion module to some extent. Thus, the whole robot system realizes a hierarchical structure from cells (jet actuators) to tissues (motion modules) and further to organs (modular robot systems). This can increase the diversity of the motion modules in structural design and the motion schemes of the robot system combined with the different types of motion modules.

With respect to the functional modules, the experiments with transportation and image‐capturing modules are conducted, while more functional modules with various shapes and sizes can be developed for the robot system, as shown in Figure S8 (Supporting Information). Due to the peculiarity of the underwater environment, the design of the functional modules can be flexible, where the size and weight are not strictly limited. The requirement is the matching between the weight and buoyancy force of a single functional module, which can be solved by adjusting the counterweights. Then, the situation is simplified to the consistency of the coupling interface for the functional module, which increases the flexibility of the module design. Besides, the functional modules can be upgraded independently and further serve in the whole robot system, indicating the upgrading characteristics of the system.

The modular reconfigurable underwater robot system shows superior motion capability and reconfiguration characteristics; however, there are still some challenges that need to be addressed for further application. First, due to the size restriction of the single motion module, its endurance is not long enough, and it cannot have the ability to work on its own. Therefore, cooperation with a water surface platform, which can ensure a complicated operation for a long time, is considered in subsequent research for specific applications. The endurance of the motion module and the distance from the platform are monitored in real‐time. Thus, a recovery signal is sent when the endurance is low, and then the module can start its own recovery and re‐charging. In addition, the buoyancy adjustment strategies have matured currently; then, a separate buoyancy or rechargeable module can be adopted to configure the buoyancy precisely and realize a rescue of the motion modules. Second, the robot system adopts permanent magnets for the coupling mechanism, which prohibits the realization of self‐reconfiguration. A connection strategy by the electromagnetic connection method cooperating with the position and attitude sensors can be adopted. Due to the difficulty of depth inspection, the reconfigurations of modules can be achieved at the surface. According to the self‐aligning ability of the magnets, it will be able to realize automatic connection by adjusting the distance between different modules. Considering that the multiple motion modules will cooperate with a platform on the water surface, the auxiliary devices, such as robotic arms, can be assembled on the platform and assist in the connection of modules.

In future work, the modular robot system will be improved in the motion modules, the specific functional modules, and the control strategies. The motion modules with more integrated sensing ability and better motion characteristics can be developed; and various functional modules with more abundant functions will be fabricated, such as water quality sensing and sound acquisition. By cooperating with the water surface platform, the robot system is expected to accomplish a series of related tasks in the area of aquatic environment with the advantage of reconfiguration and replacement, from environment exploration to data acquisition, and to long‐time inspection in the designated narrow spaces.

## 8 Experimental Section

### Materials and Fabrication of Motion Modules

The motion modules were all fabricated by Stereolithography Appearance (SLA) 3D printing. The materials of the middle frame, the motor base, and the jet cavity structure were selected as 9000R Ultraviolet (UV) Curable Resin, with a flexural modulus of ≈2700 MPa and a density of ≈1.15 g cm^−3^. The transparent cover was made of 8001 transparent UV Curable Resin material, whose light transmission rate can reach 90.8%. The material of the thin plate of the jet actuator was selected as polyvinyl chloride (PVC) plastic. The different external structures were sealed by waterproof adhesive, while the jet cavity and the thin plate were connected by transparent epoxy adhesive. All motion modules have identical dimensions of 48 mm × 38 mm × 38 mm, and the overall mass was ≈79 g.

### Experiment Design of Flow Field Observations

A particle image velocimetry (PIV) method was used to observe the flow field produced by the jet actuator. The experimental environment was set in a shaded dark chamber with a green laser source for illumination. The tracer particles were specialized Polyamide Resion Particles (PSP) with a diameter of ≈20 µm, a refractive index of 1.56, and a particle density of 1.03 g cc^−1^, which ensured that the particles would not deposit in water. A high‐speed charge coupled device (CCD) camera (DAHENG MER‐031‐860U3M) was utilized for image capturing, and its frame rate can reach 860 fps with a resolution of 640 × 480. The surfactant was utilized to guarantee that the particles could be distributed evenly in water. A jet actuator was specially designed to facilitate clamping underwater, and a direct current (DC) regulated power supply (UNI‐T UTP1306‐II) was used to power the ERM motor.

### Software Development of the Control System

The modular reconfigurable robot system uses Bluetooth Low Energy (BLE) unit to realize wireless communication. Aiming at the modular robot system, a control software with one‐to‐many Bluetooth connectivity was specially developed based on the Android studio with Java language (see Figure S4b, Supporting Information). This software can realize the connection with different motion modules simultaneously by storing the information of all the BLE devices. Then, it can give control commands to selected modules individually according to the motion requirements, thus reducing the control difficulty of the multi‐module combined robot system.

### Rotary Speed Measuring Experiment

A non‐contact tachometer (UNI‐T UT372) was used to measure and calibrate the rotary speed of the waterproof ERM motor under different voltages. The measurement range of rotatory speed was 10–99 999 RPM with an accuracy of ± (0.04%+2). Twenty values of the rotary speed are obtained at each voltage, and the experimental setup is shown in Figure S12a,b (Supporting Information). A DC‐regulated power supply (UNI‐T UTP1306‐II) was used to supply power to the ERM motor, and the measured voltage range was set from 0 to 3.3 V. The rotary speed–voltage curve of the ERM motor is shown in Figure S12c (Supporting Information), in which the maximum excited frequency was ≈270 Hz.

### Design of Functional Modules

A transportation module and an image‐capturing module are designed as functional modules, respectively. The main structures of both functional modules are fabricated by the SLA 3D printing method and connected to the motion modules by permanent magnets. The containers of the modules were made of 9000R UV Curable Resin, while the covers were made of 8001 transparent UV Curable Resin material. The transportation module adopts a cubic shape, which can connect with the motion modules in both diagonal and linear configurations. The overall size was 32 mm × 38 mm × 38 mm, and the overall mass was ≈21 g. Then, this module was expected to carry an object with a mass of ≈25 g. The image‐capturing module arranges the coupling interface on the adjacent and perpendicular faces, which can realize diagonal configuration with the motion modules. The size of the contact surface on the image‐capturing module was 38 mm × 32 mm, and the total mass was ≈30 g. The video acquisition function was realized by adopting the ESP32 as the microcontrol unit and the OV2640 as the image sensor. A wireless image transmission was realized by a Wi‐Fi connection with the host computer; the video can also be stored in the Micro Secure Digital (SD Card) directly.

### Motion Performance Measurement Method

An aquatic robot motion platform was constructed in the laboratory. A water tank with dimensions of 100 mm × 70 mm ×40 mm was built underneath by acrylic plates, and cutting mats are arranged inside the tank to serve as motion scales. A digital camera (HIKVISION E14a) was set on the top and connected to the computer to acquire the motion videos of the robot system. The motion videos of the robot system were processed using the frame superposition method during post‐processing, so as to obtain the motion velocity parameters of the robot system.

### Statistical Analysis

The sample size (*n*) for each statistical analysis was *n* = 3. The data were expressed as mean ± SD (Standard Deviation). Statistical analysis of the data was performed using OriginPro 2024.

## Conflict of Interest

The authors declare no conflict of interest.

## Supporting information

Supporting Information

Supplemental Movie 1

Supplemental Movie 2

Supplemental Movie 3

Supplemental Movie 4

Supplemental Movie 5

Supplemental Movie 6

Supplemental Movie 7

## Data Availability

The data that support the findings of this study are available from the corresponding author upon reasonable request.
