# Supplementary material for: Miniature Modular Reconfigurable Underwater Robot Based on Synthetic Jet
Source: Adv Sci (Weinh). 2024 Aug 13;11(39):2406956. doi: 10.1002/advs.202406956 (PMC11496987; doi:10.1002/advs.202406956)
Supplement: Supplementary file 1 — Supporting Information [file ADVS-11-2406956-s002.docx]

Supporting Information

**Miniature modular reconfigurable underwater robot based on synthetic jet**

*Dehong Wang, Fanheng Zhang, Shijing Zhang, Daqing Liu, Jing Li, Weishan Chen, Jie Deng*, Yingxiang Liu**

Email: dengjie21@hit.edu.cn (J. D.), liuyingxiang868@hit.edu.cn (Y.L.).

**The PDF file includes:**

Supplementary Note S1 to S7

Figure S1 to S12

**Other Supporting Information includes the following:**

Movie S1 to S7

**Supplementary Note**

Note S1. Buoyancy design of the motion module in water

For an underwater robot, the balance between its buoyancy and gravity is significant for stable motion. Therefore, we focus on the buoyancy-gravity relationship of the robot when designing the structure of a single motion module, as shown in Figure S3a-b. According to the calculation of the mass and position distribution of each component, the overall mass of a motion module is about 78 g (with a real mass of 79 g by considering the influence of the wires and the waterproof adhesive, see Figure S3c), and its mass center is located at 18.27 mm above the bottom surface. The displaced water volume of the robot shows that the mass of the displaced water is about 80 g with a buoyancy force of about 0.78 N. The buoyancy center is about 19.22 mm above the bottom surface. It can be seen that the mass center of the robot is located under the buoyancy center, which ensures that the motion module can be stabilized in underwater environments. When the motion module is placed in the water, the module is almost in a suspended state, as shown in Figure S3d. Similarly, the combined dual motion modules are also in a suspended state, as shown in Figure S3e. This ensures that the motion modules can effectively realize the motion in the underwater environment.

Note S2. Design of the control system of the motion module

To guarantee the independently controllable motion capability for each motion module of the robot system, a power-control-communication electronic system is completely integrated inside each module. The basic frame is shown in Figure S4. The whole system can be divided into three layers from the top to the bottom. The top layer is the remote-control software, which can realize the simultaneous control of multiple motion modules by the mobile terminal, as shown in Figure S4a-b. The middle layer is the control part, which adopts a BLE (Bluetooth low energy) unit to realize communication with the remote-control software and utilizes an MCU (Micro-control unit) to realize the regulation of the motor rotary speed, as shown in Figure S4c. The bottom layer is the actuation part, which mainly contains the motor driver units to excite the rotation of ERM motors, as shown in Figure S4d. The middle and bottom layers are integrated into the motion module, and a battery and a voltage regulator unit are used to power both layers simultaneously.

The software is designed based on the Android platform. It can realize two-way communication with multiple modules simultaneously. A specialized communication protocol is designed to ensure the stability of transmission. The UI of the remote-control software is shown in Figure S4b. In the connect panel, the desired motion module to be connected can be selected, while in the control panel, the control commands can be sent to different selected modules. For the integrated layers, the middle and bottom layers are designed into the control and actuation boards separately. In the control board, the STM32F103 is used as the micro control unit, and the HL-B40 is used as the communication unit. In the actuation board, the DRV8833 motor driver is used, and the output voltages can be regulated by the PWM method. The power supply adopts a 200 mAh rechargeable lithium battery, and a DC-DC voltage regulator module is utilized for the stable voltage output of 3.3 V.

In addition, the image-capturing module uses an ESP32-S3 MCU with an OV2640 sensor to realize wireless transmission with the remote software, and a software is developed based on C# on the computer. It can realize the stream transmission of the image based on UDP protocol by Wi-Fi. Thus, a real-time modification and acquisition of the images can be achieved.

Note S3. Control methods for motion orientation of single motion module.

The linear motion is the basis for the motion module to realize a controllable motion, and the consistency of the propulsion effect between the side jet actuators is essential for a linear motion. For a single motion module, we utilize two methods to guarantee consistency in this work. Firstly, the rotary speeds of the motors on the two side jet actuators are evaluated in the design of the single motion module based on the Fourier transform of the audio signals. It guarantees the proximity of the excitation frequency as much as possible, thus ensuring the consistency of the driving effect on both sides. Secondly, the excitation voltages of the two side jet actuators can be fine adjusted by the PWM method during the motion state, thus adjusting the excitation frequencies, as illustrated in Note S2. Then, the linear motion will be modified by an open-loop control method in real-time. The PWM method is more suitable for the cases of multi-module systems, and more jet actuators can be adjusted to ensure linear motions in different directions.

As for the actual motion orientation, it can be adjusted by controlling the two side jet actuators individually for a single motion module. The direction will be regulated relatively precisely at a slower speed by the PWM adjustment as well as with the assistance of the reverse compensation from the other side jet actuator. The propulsion effect for linear motion will be enhanced afterward by re-increasing the excitation voltages. Besides, for the multi-module combined system, the orientation can be modified more conveniently. The redundant jet actuators can be excited to realize a real-time adjustment without affecting the previous excited jet actuators.

Note S4. Connection reliability evaluation of the modular robot system.

The connection reliability is essential for the modular robot system to realize a stable motion in the aquatic environment. Therefore, we have conducted related experiments to evaluate the connection state between the motion modules. A digital push-pull force gauge (HANDPI HP-500) is utilized with an accuracy of 0.1 N. The magnets between two motion modules can provide a force of about 2.3 N in the axial direction and a force of about 2.0 N in the tangential direction. It means that if the influence of external disturbance cannot exceed this magnetic force, the connected modules can ensure a stable motion without separation in the actual movement.

In this work, we mainly investigate the motion performance of the modular robot system in the still water environment, and no separation occurs during the experiments. Here, we take the dual-module case as an example to analyze the force in the motion process. When the module produces a linear motion under the excitation of the side jet actuators SJA_1L_ and SJA_2R_, a drag force is applied on the front surface. Then, the separation situation may happen due to the torque produced by the propulsion and drag forces, which is less than 20 mN. It is much smaller than the magnetic force. Besides, the two side jet actuators SJA_1R_ and SJA_2L_ will further prohibit this separation.

The pressure caused by the water depth is hydrostatic pressure, which acts in all directions and will not affect the connection between the modules directly. Thus, a stable module connection can be ensured when moving in still water.

Note S5. The other configurations of the motion modules

We mainly showcase the cubic intermediate modules with the same size as the motion modules. However, the structure design of the intermediate modules can be flexible, and the universal coupling interface also guarantees the adaptability of the motion modules for combination. Therefore, some other configurations of the motion modules combined with various intermediate modules in different shapes are shown in Figure S8.

For the rectangular functional modules, three types of combination methods can be designed, as shown in Figure S8a. The motion modules can be arranged on the same side or on the two sides of the functional module in the length direction. They can also be set on the two ends of the module. As for the triangular functional modules with different angular characteristics, we can also adopt the configuration modes corresponding to different angles, as shown in Figure S8b. This ability to combine with the functional modules in different shapes ensures its application potential. Further, the functional modules with more edges can also be connected to the motion module directly, as shown in Figure S8c, except the modules with smooth surfaces, which can be handled by auxiliary modules.

Note S6. Linear configurations with intermediate modules

When dual motion modules are used to combine with the intermediate modules, there exists a typical linear configuration, as shown in Figure S10a, except for the diagonal configuration shown in Figure 7. This configuration sandwiches the intermediate module in the middle to form a line structure. In contrast to the diagonal arrangement, it can only achieve forward linear motion when the side jet actuators SJA_1L_ and SJA_2R_ are excited simultaneously. When the side jet actuators SJA_1L_ and SJA_2L_ are excited, it can produce clockwise rotation and when SJA_1R_ and SJA_2R_ are excited, it will produce counterclockwise rotation, as shown in Figure S10b. This configuration will have two speed stages in bi-directional rotational motions when each of the side jet actuators is excited independently.

Then, we replace the intermediate module with a transportation module to conduct experiments, as shown in Figure S10c. The robot system can realize clockwise rotational motion with a speed of about 1.1 rad s^-1^, and counterclockwise rotational motion with a speed of about 1.0 rad s^-1^, as shown in Figure S10d-e. The robot system can realize the linear motion with a speed of 55.4 mm s^-1^, as shown in Figure S10f. In addition, due to its elongated structural characteristic, this configuration can be modified to allow a single jet actuator to pass through the geometrical center of the robot system. Thus, it can achieve the passing motion through a narrow slit, as shown in Figure S10g.

Note S7. Motion performance comparison with different aquatic robots.

The common motion strategies for underwater locomotion include the traditional propeller method, the body or caudal fin (BCF) propulsion method, the median and paired fin (MPF) propulsion method, the jet propulsion method, and the turtle-like method, etc. The realizations of these methods are also complicated, such as the mechanical mechanism, origami, pneumatic, piezoelectric, dielectric elastomer actuators (DEA), shape memory alloy (SMA), ionic polymer–metal composite (IPMC), and phototactic materials. We investigate the research extensively containing the above methods, as shown in Figure S11. The figure shows the traditional propeller thrust, the BCF propulsion, the MPF propulsion, the jet propulsion, and other typical propulsion methods with square, circle, diamond, down triangle, and up triangle symbols respectively. The hollow symbols indicate that the prototypes have not realized the integration of the power supply and control system. It can be seen that the motion module in this work exhibits superior motion performance in terms of both size and motion speed among the other underwater robots. The relative motion speed of this prototype outperforms the majority of existing robots, except for a few mature BCF robots with larger dimensions (larger than 200 mm, such as Ref 44,49,51,68). The speed characteristic of the prototype is more distinct when only the integrated robots are considered. The motion module achieves high integration and fast motion speed at the same time in such a small size, which guarantees the mobility of the whole modular robot system and also proves the profound potential for subsequent research.


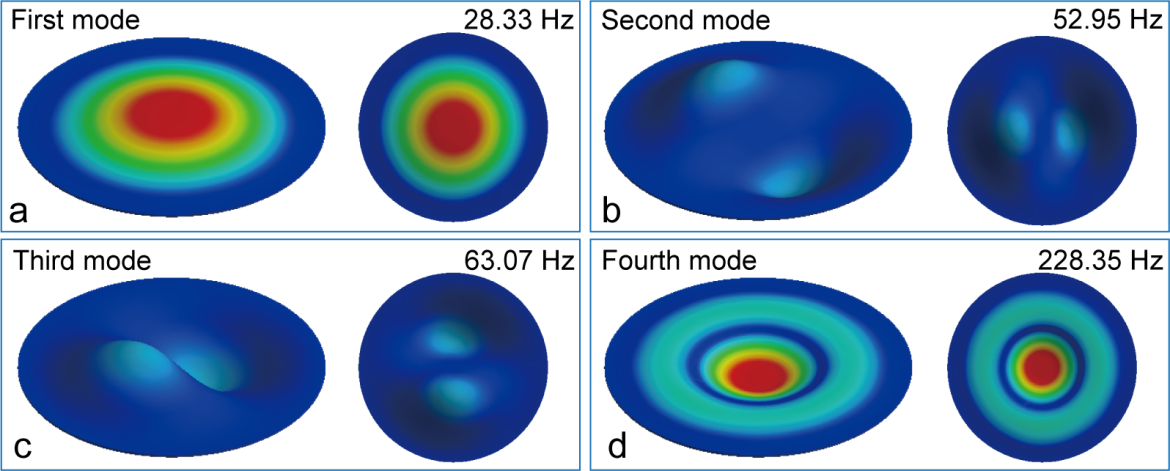


Figure S1. The mode shapes of the thin plate under excitation. a) The first mode shape at 28.33 Hz. b) The second mode shape at 52.95 Hz. c) The third mode shape at 63.07 Hz. d) The fourth mode shape at 228.35 Hz.


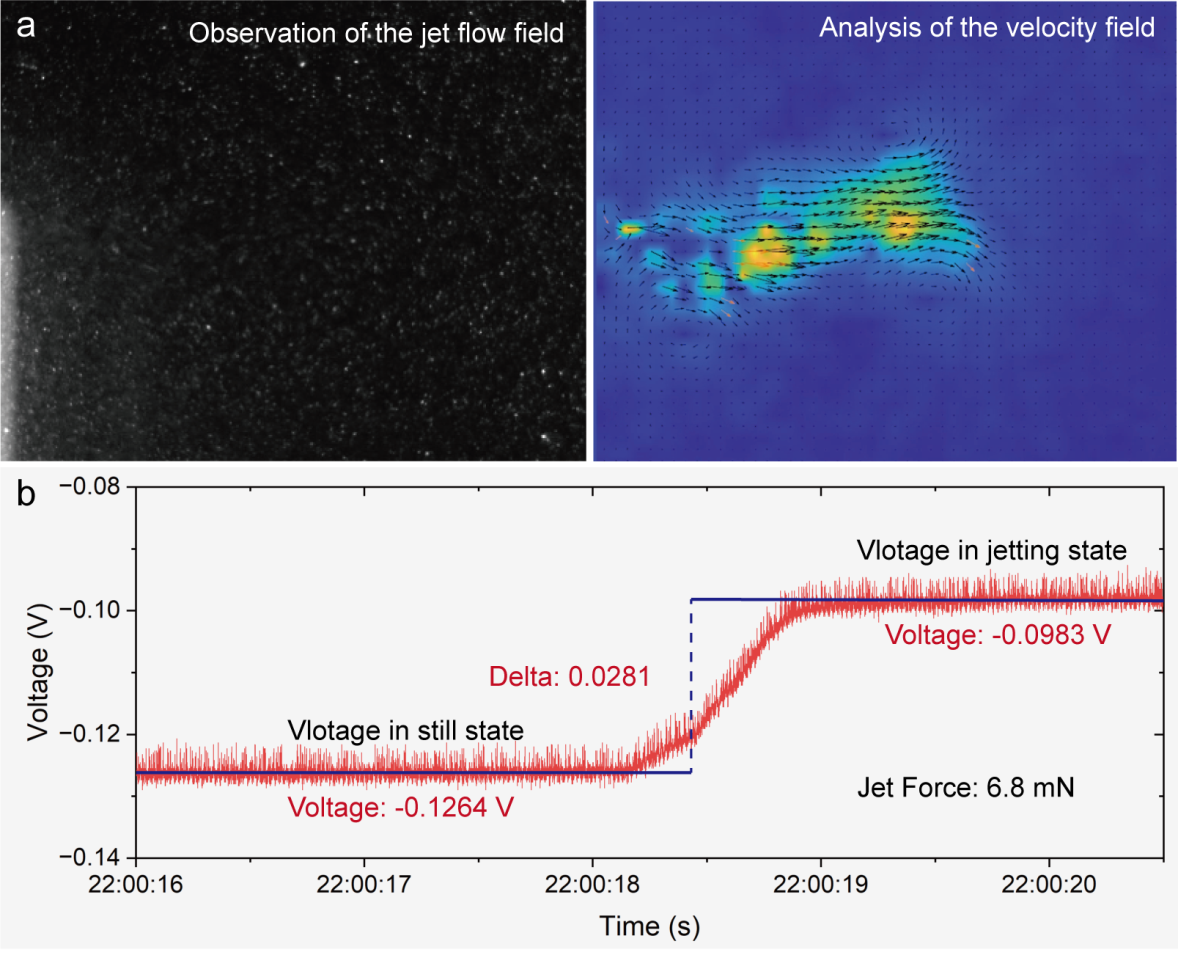


Figure S2. Flow field parameters of a synthetic jet actuator. a) The flow field observed by the PIV method. b) The propulsion force produced by a synthetic jet actuator.


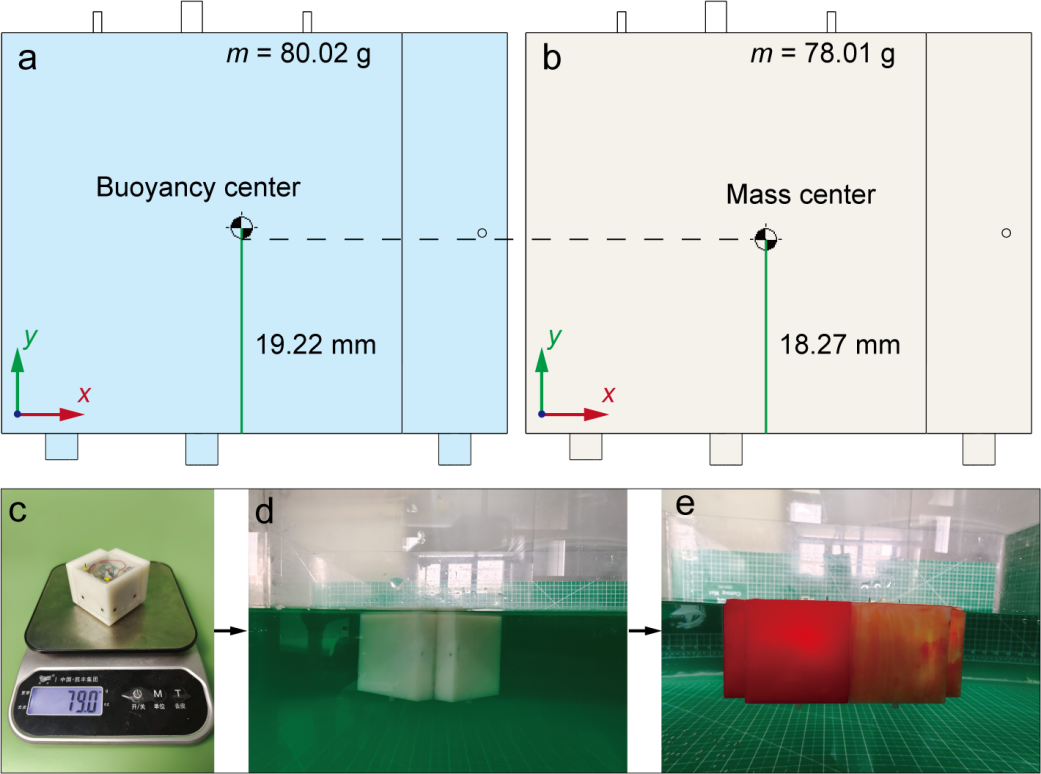


Figure S3. Buoyancy design of the motion module. a) Position of the buoyancy center. b) Position of the mass center. c) Real mass of a single motion module. d) Suspension of single motion module in water. e) Suspension of dual motion modules in water.


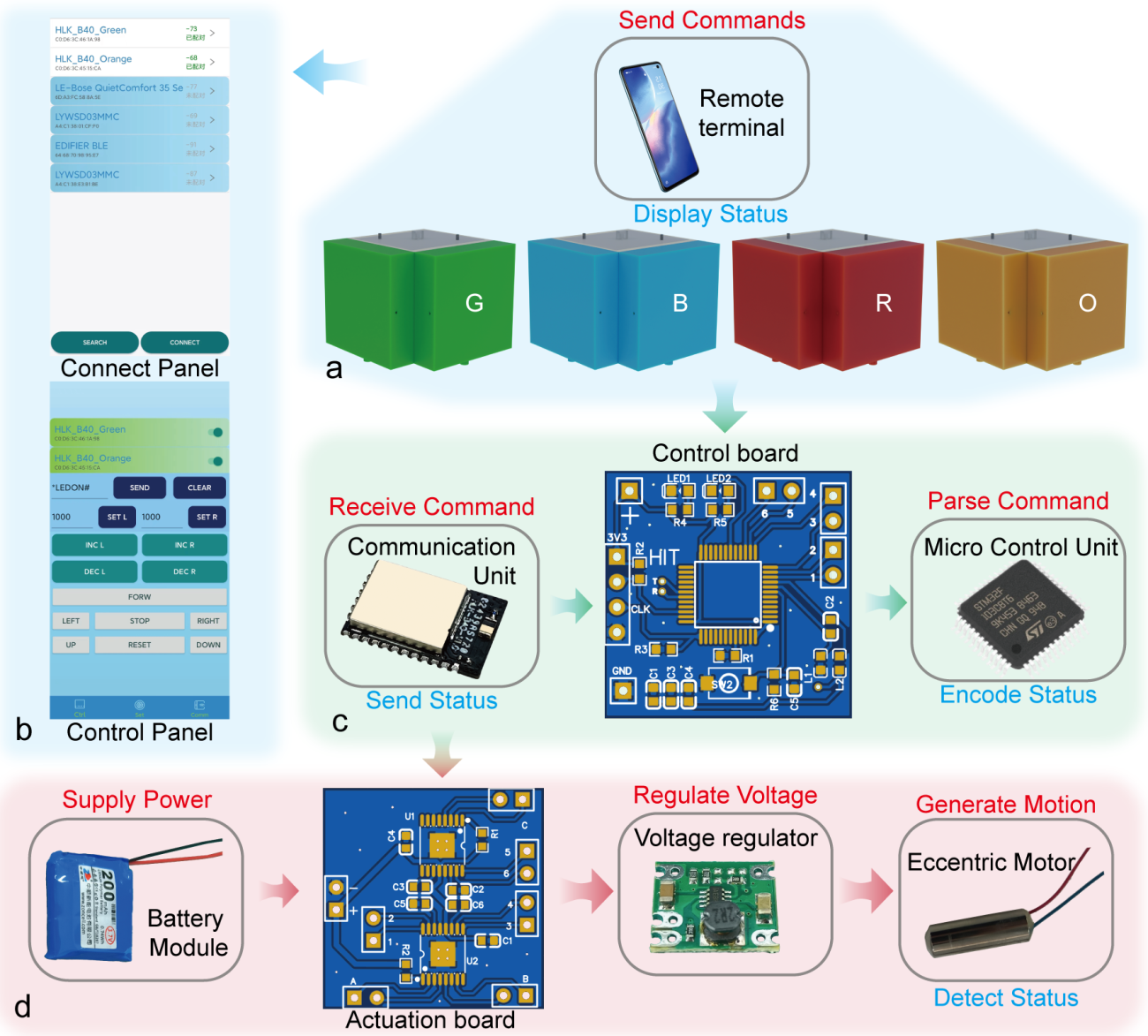


Figure S4. The framework of the control system of the module reconfigurable robots. a) 1-to-N remote-control software of the control system. b) The UI design of the software. c) The control and communication level of the control system. d) The power supply and actuation level of the control system.


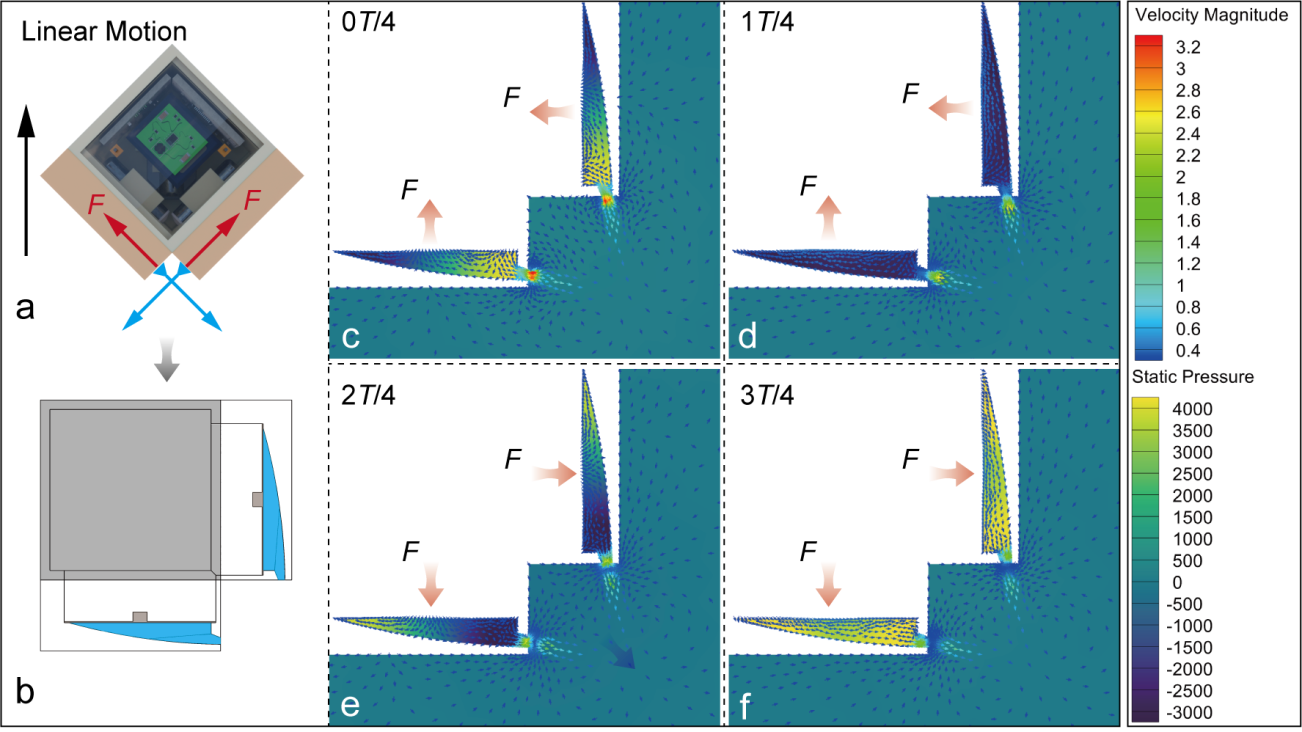


Figure S5. Fluid-structure interaction simulation for linear motion case. a) Linear motion scheme of the motion module. b) Inner structure of the dual side jet actuators. c) Simulated flow fields at 0*T*/4 time. d) Simulated flow fields at 1*T*/4 time. e) Simulated flow fields at 2*T*/4 time. f) Simulated flow fields at 3*T*/4 time.


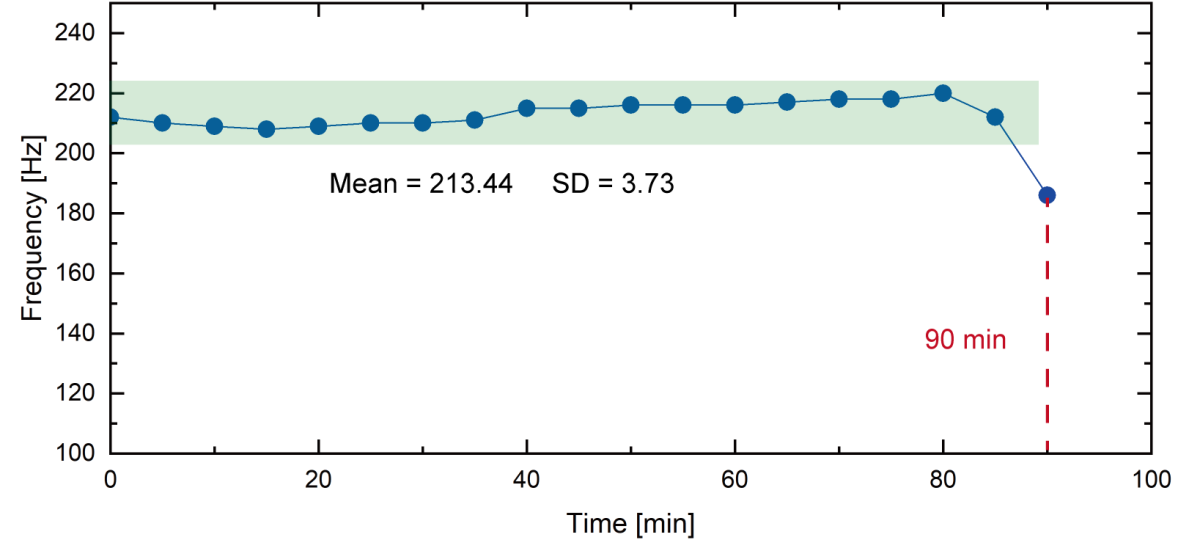


Figure S6. Endurance evaluation of the single motion module when a single jet actuator is excited.


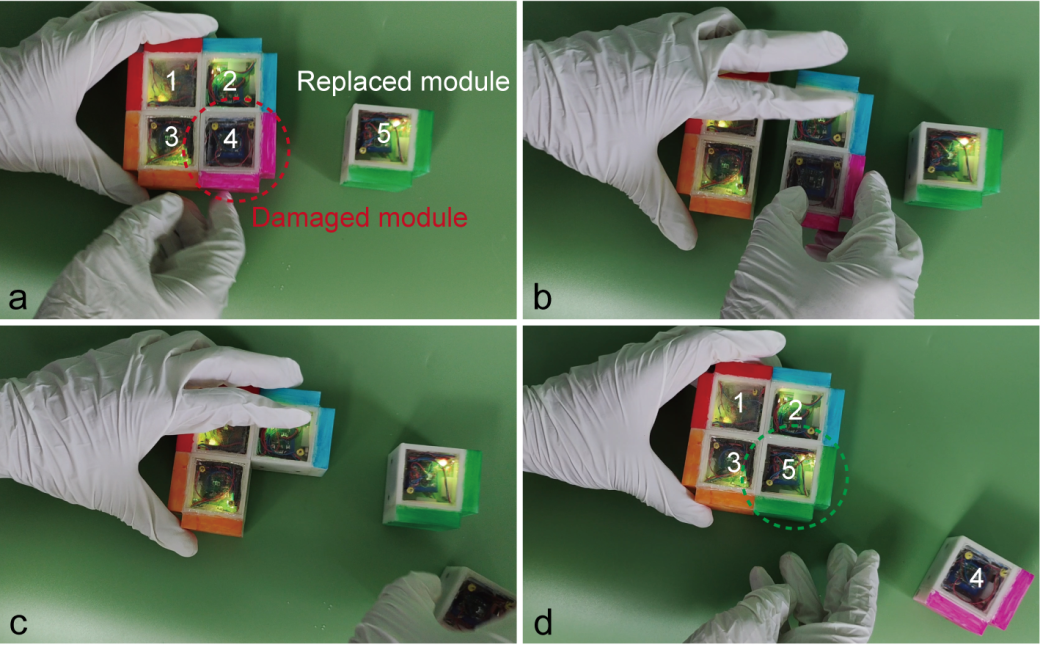


Figure S7. The process of the replacement of the motion module. a) The preparation of the replaced module. b) The disassembly of the combined modules. c) The removal of the damaged module. d) The replacement of the damaged module.


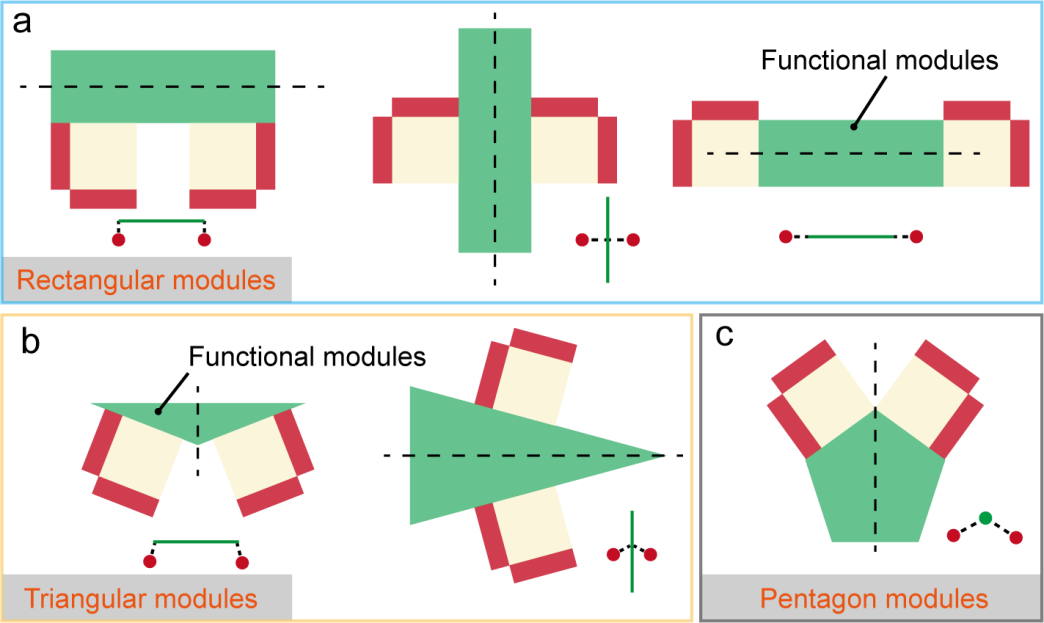


Figure S8. Various configurations when combined with different intermediate modules. a) The configurations with rectangular functional modules. b) The configurations with triangular functional modules. c) The configurations with pentagon functional modules.


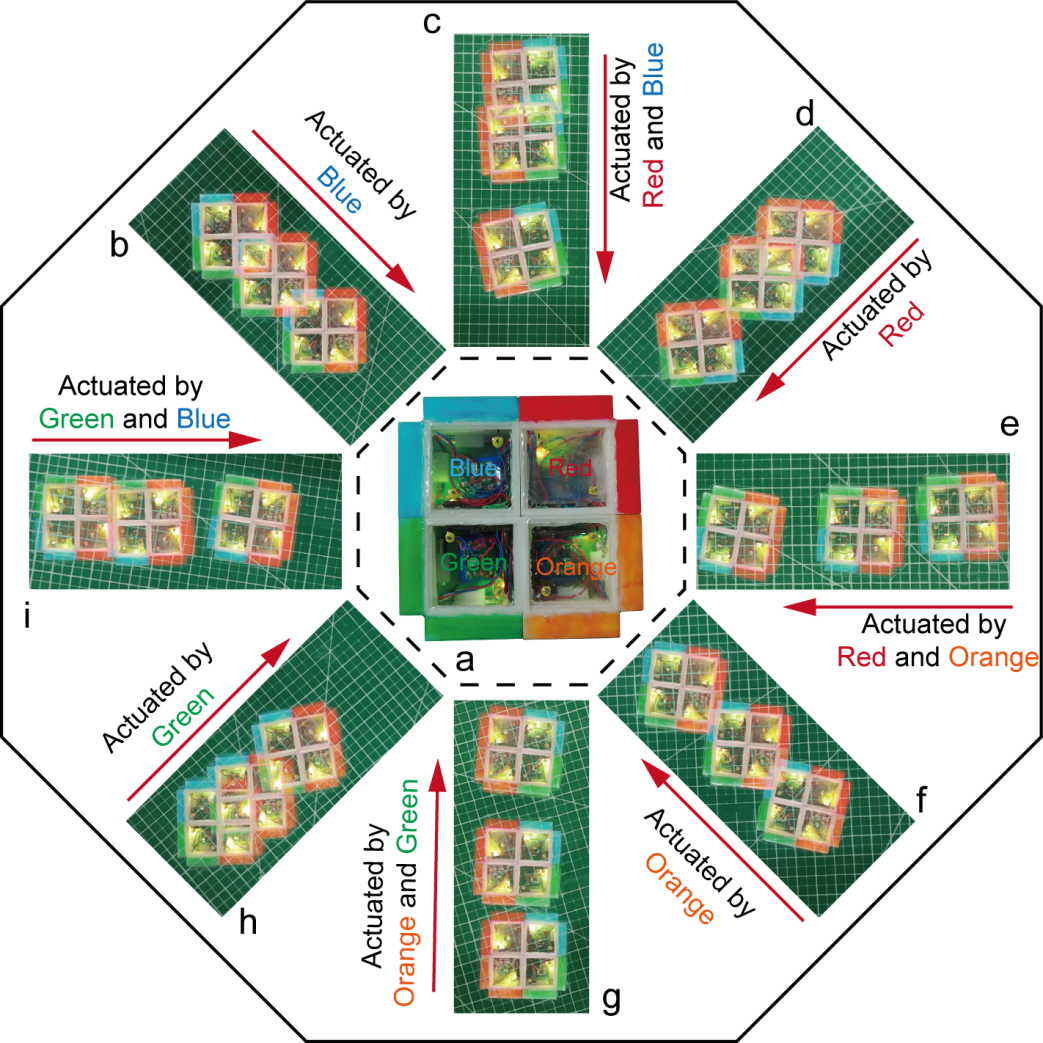


Figure S9. The linear motion schemes of quad motion modules. a) Quad motion module combined robot system. b) The linear motion actuated by the blue module. c) The linear motion actuated by the blue and red modules. d) The linear motion actuated by the red module. e) The linear motion actuated by the orange and red modules. f) The linear motion actuated by the orange module. g) The linear motion actuated by the orange and green modules. h) The linear motion actuated by the green module. i) The linear motion actuated by the blue and green modules.


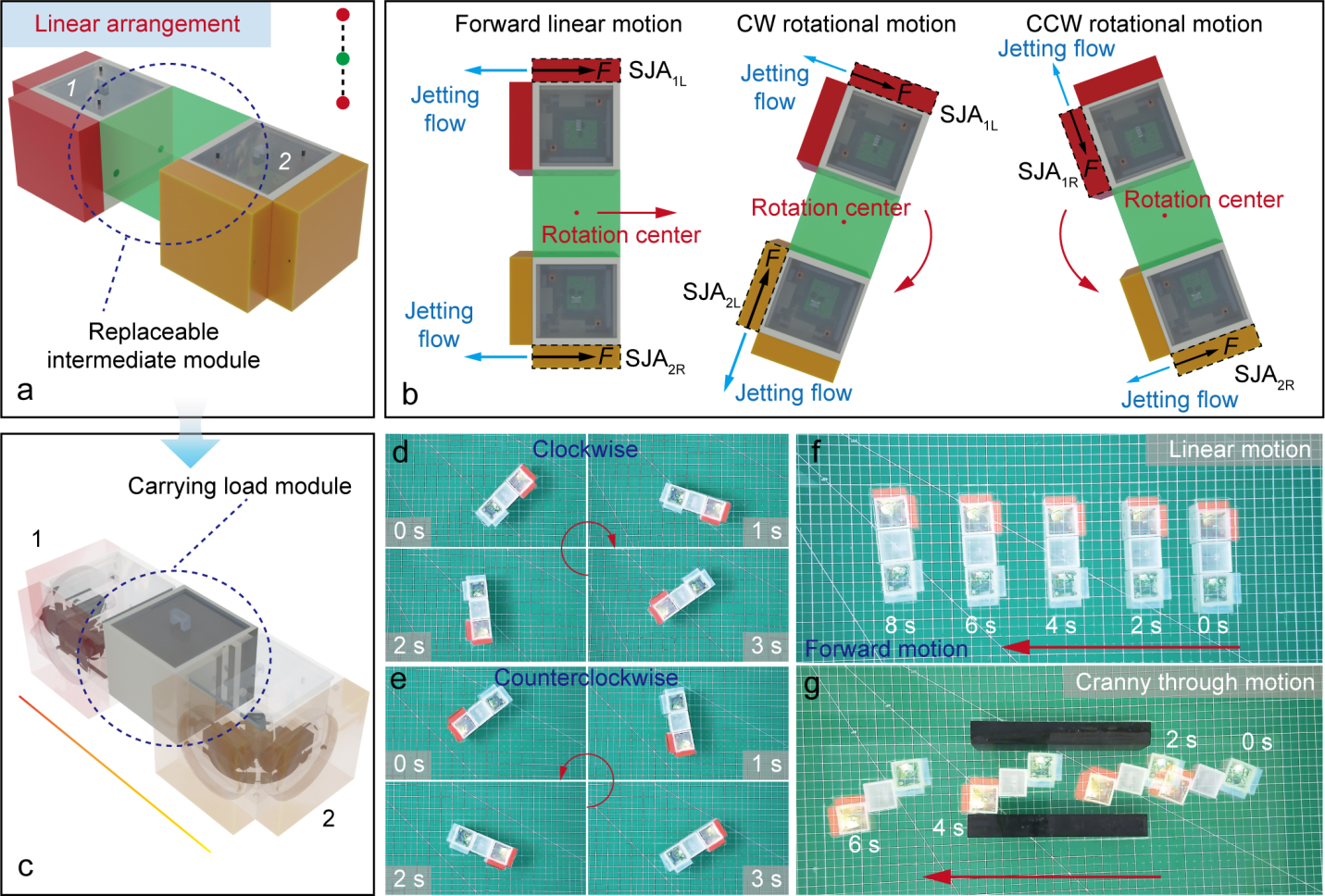


Figure S10. Motion scheme and experiments of linear configuration. a) Linear configuration with intermediate module. b) Basic motion schemes of the linear configuration. c) Linear configuration with transportation module. d) Clockwise rotational motion of the robot configuration carrying a 20 g weight. e) Counterclockwise rotational motion of the robot configuration carrying a 20 g weight. f) Linear motion of the robot configuration carrying a 20 g weight. g) Motion through the narrow channel by a modified robot configuration carrying a 20 g weight.


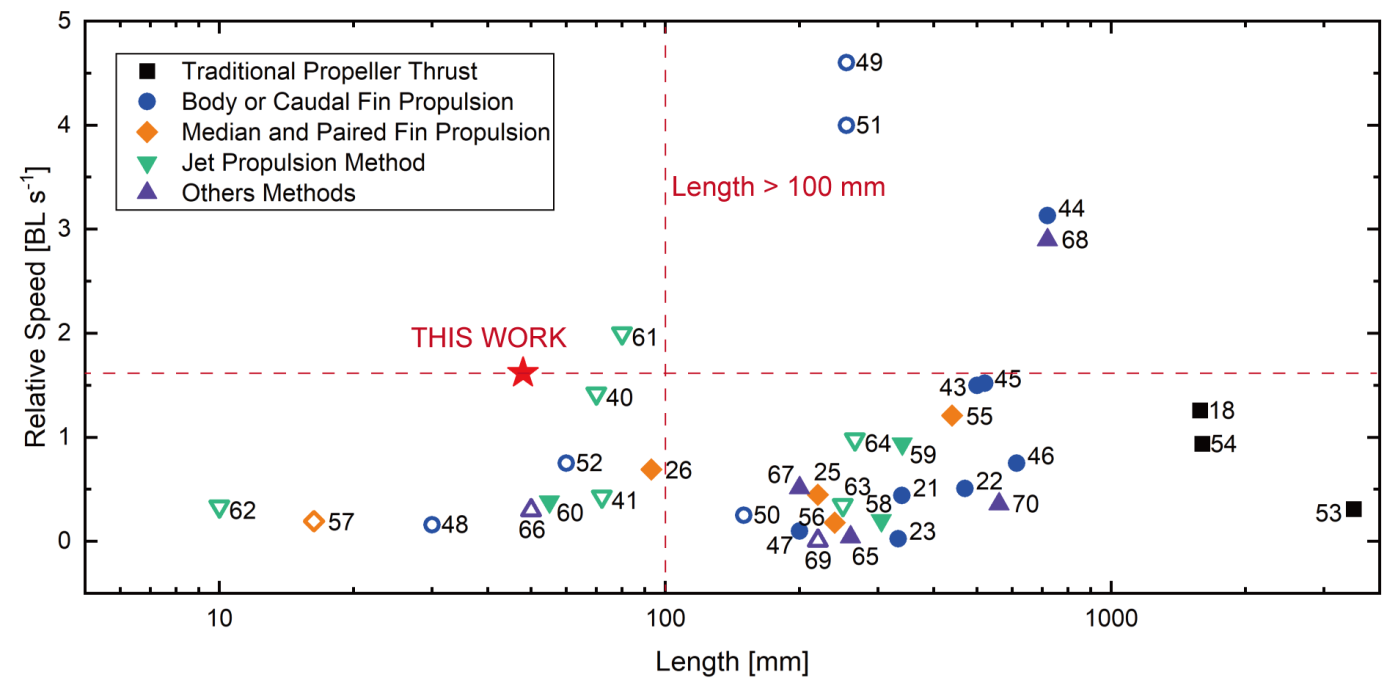


Figure S11. Motion performance comparison with other different aquatic robots.


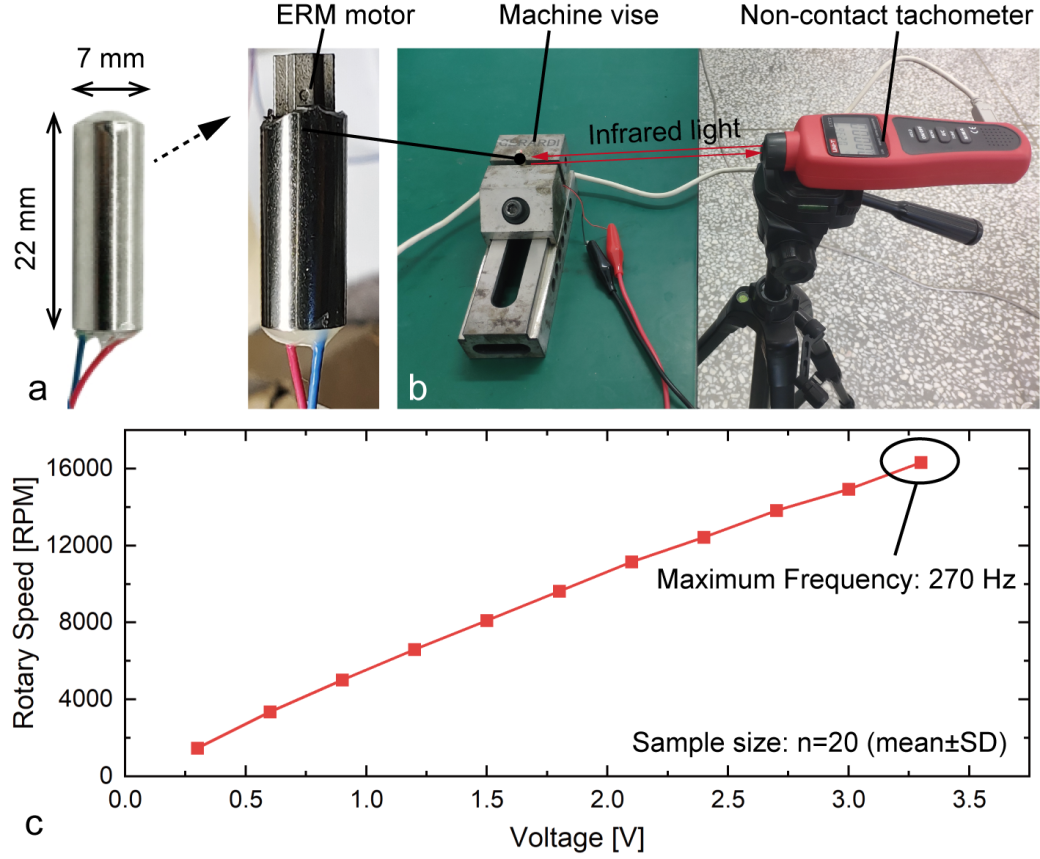


Figure S12. Experiment design of the rotary speed measurement of the ERM motor. a) The size of the waterproof ERM motor. b) Rotary speed measurement of the ERM motor. c) Rotary speed of the ERM motor with different voltages (Sample size n=20, Mean ± SD).

Movie S1.

Motion schemes of the modular robot system.

Movie S2.

Evolution process of synthetic jet flow field.

Movie S3.

Experiments of single multi-DOF motion module.

Movie S4.

Motion experiments of dual motion modules.

Movie S5.

Motion experiments of quad motion modules.

Movie S6.

Experiments of corner configuration with functional modules.

Movie S7.

Experiment of line configuration with functional modules.
